# Surface Acoustic Wave Mitigation of Precipitate Deposition on a Solid Surface—An Active Self-Cleaning Strategy

**DOI:** 10.1021/acsami.1c17778

**Published:** 2021-12-01

**Authors:** Yifan Li, Dario R. Dekel, Ofer Manor

**Affiliations:** † The Wolfson Faculty Department of Chemical Engineering, Technion—Israel Institute of Technology, Haifa 3200003, Israel; ‡ The Nancy & Stephen Grand Technion Energy Program (GTEP), Technion Israel Institute of Technology, Haifa 3200003, Israel

**Keywords:** surface acoustic wave, self-cleaning surface, coating, precipitation, liquid film, convective self assembly

## Abstract

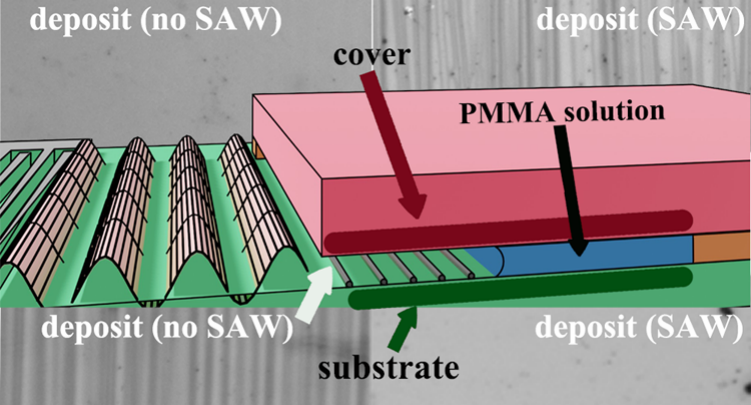

We demonstrate the application of a 20 MHz frequency surface acoustic wave (SAW) in a solid substrate to render its surface “self-cleaning”, redirecting the deposition of precipitating mass onto a nearby inert substrate. In our experiment, we confine a solution of poly(methyl methacrylate) polymer and a volatile toluene solvent between two substrates, lithium niobate and glass, at close proximity. We render the glass surface low energy by employing hydrophobic coating. In the absence of SAW excitation, we observe that the evaporation of the solvent yields polymer coating on the higher energy lithium niobate surface, while the glass surface is mostly devoid of polymer deposits. The application of a propagating SAW in the lithium niobate substrate mitigates the deposition of the polymer on its surface. As a response, we observe an increase in the deposition of the polymer precipitates on glass. Above a SAW power threshold, the polymer appears to deposit solely on glass, leaving the surface of the lithium niobate substrate devoid of polymer mass.

## 1 Introduction

The origin of this work is connected to many applications that employ liquid flow in ducts, pipes, or atop open surfaces for heat and mass transport or for chemical and mechanical fabrication of products. Familiar examples to most are domestic utilities, such as water heaters, dishwashers, and washing machines. In time, the utilities become covered by layers of minerals, particularly calcium carbonate, which precipitate out of volatile tap water.^1^ Other examples include industry-based unit operations, especially in the oil industry and fabrication processes, which may be associated with precipitates from organic solvents.^2,3^ Common methods to eliminate the precipitation of mass from a solvent are to render the solute more soluble. In the case of water, this is associated with the addition of chemical water softeners or using ion exchangers, replacing precipitating hard salts with softer counterparts, which possess greater tendencies to stay in their ionic state in the solution.^4^ Alternatively, the use of ultraclean solvents is especially common for microelectronic fabrication. The former solution to the problem yields mineralization in case that the carrier liquid evaporates. The latter solution to the problem requires high-cost ultraclean solvents that are often corrosive, for example, deionized (DI) water. A different option to keep surfaces clean is to employ self-cleaning surfaces, which may avoid or easily get rid of undesirable deposits.

Passive self-cleaning surfaces have been studied for a long time. The studies have mostly concentrated on superhydrophobic and superoleophobic surface properties.^5−7^ The surfaces support roughness of different length scales, which trap air bubble cushions underneath sessile drops, supporting the Casi–Baxter wetting state. The latter is usually associated with a small three-phase contact angle hysteresis level. The contact angle hysteresis is approximately proportional to mechanical resistance to the motion of a drop along a surface.^8^ A tilt of the surface with respect to the horizon leads to the roll of drops, which collect particulates and dirt in their path. However, the fine surface roughness in these cases is sensitive to mechanical impact and abrasive elements. Moreover, should a drop attain the more thermodynamically stable Wenzel wetting state, where the drop attaches directly to the rough solid, the drop will stick to the solid at a greater adhesion energy than it will to a flat surface of the same chemistry. Passive solutions meant to improve the mechanical and thermodynamic stability of the abovementioned substrates are oil-infused surfaces, which further employ an additional oil layer atop the rough surface.^9^ Here, we consider a very different approach for self-cleaning surfaces by employing active mechanical stimuli on flat surfaces.

Several studies^10−13^ considered the active diminution of the three-phase contact angle hysteresis and hence active contributions to dynamic wetting effects by an oscillating external force, in particular, mechanical vibration. The physics of an active mitigation of contact angle hysteresis by oscillating external force^14^ is similar to the mechanism that reduces the effective friction between vibrating solids. The oscillating force effectively reduces friction. Above a threshold level, the oscillating force may eliminate friction. Moreover, many modern applications of mechanical vibration for generating flow and pressure fields in liquids employ MHz-frequency surface acoustic waves (SAWs). In particular, SAWs were used to provide an effective change in the apparent three-phase contact angle between water, its vapor, and an underlying substrate^15^ and generate a steady drift of liquid mass (acoustic streaming) along its path,^16^ which was found to power the dynamic wetting of oil and water films.^17−21^

Previous attempts in employing SAWs to alter mass deposition on a solid surface manipulated the deposition of solute mass from a volatile solution; the latter undergoes dynamic dewetting during the evaporation of the solvent. In the absence of SAWs in the substrate, the volatile solution provides pattern formation in the deposit, also known as convective self assembly, pattern deposition, or the coffee ring effect. The pattern formation appears due to the motion of the three-phase contact line^22−24^ and due to the precipitation of solute mass.^25−28^ The presence of a SAW in the substrate, while the latter is in contact with a volatile solution, alters the flow regime and pressure field in the solution. The SAW was further found to alter the morphology of the solute deposits on the substrate following the full evaporation of the solution,^29,30^ albeit in these previous studies, the SAW was not found to eliminate the deposit itself. Above a threshold acoustic power, the SAW was found to provide homogeneous coating of the substrate by solute mass.

In this study, we demonstrate an active self-cleaning flat surface of a substrate that supports a MHz-frequency SAW. The substrate, which is in contact with a volatile polymer solution, prevents polymer precipitates from attaching to its surface. We describe our experimental procedure in Experimental Methods, highlight our findings in Results, discuss and conclude our work in Discussion and Conclusion, and give experimental details in Experimental Methods.

## 2 Results

**Figure 1:**
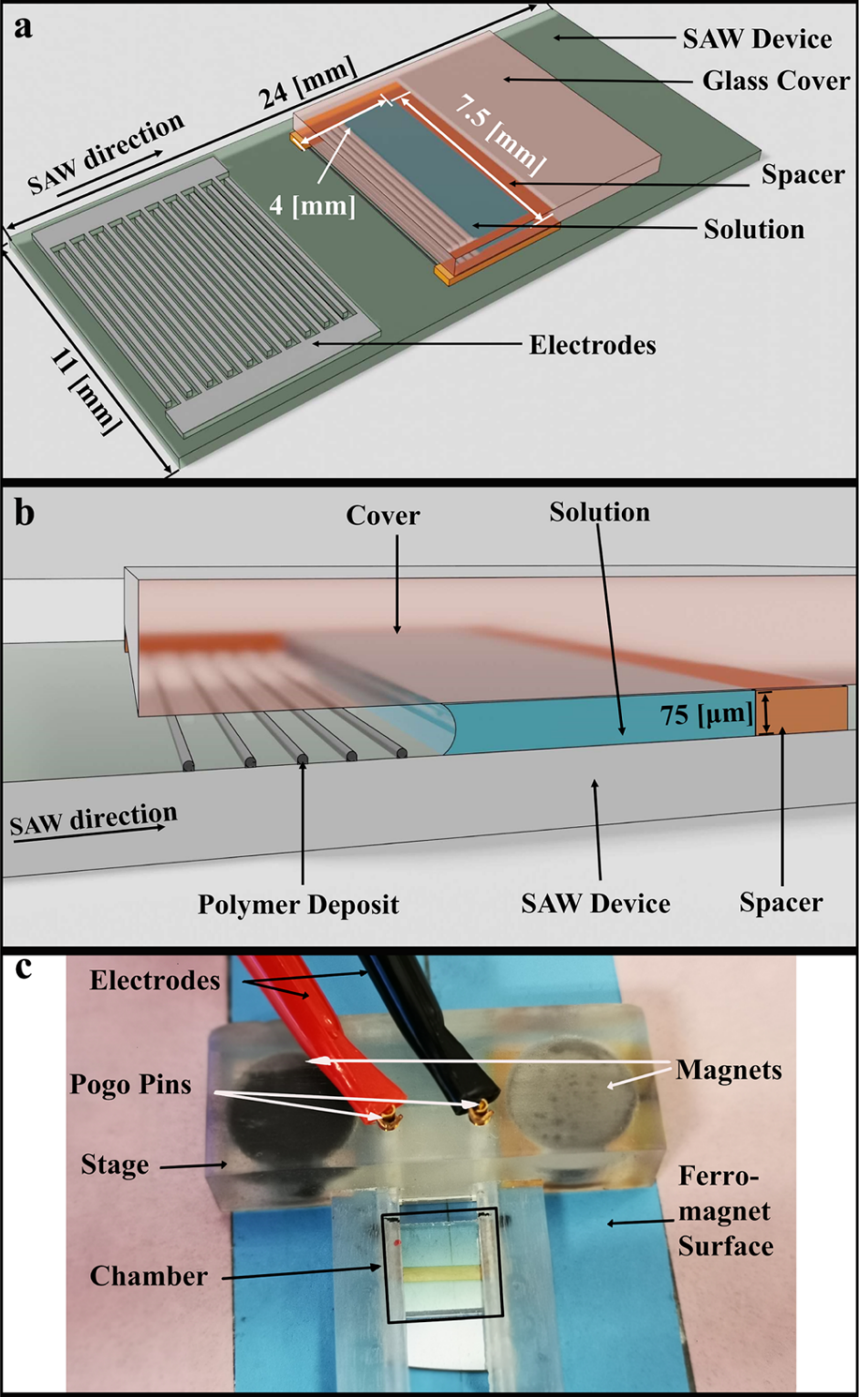
(a) Upper and (b) side views of an illustration of a microchamber, which is fabricated atop a SAW device and contains a volatile PMMA/toluene solution, where the evaporation of the latter leaves patterned deposits of PMMA in the chamber and (c) image portraying a view from above of the integrated microfluidic platform (chamber + actuator + power connection) on a dedicated stage.

We employ a 20 MHz-frequency Rayleigh type SAW to mitigate the deposition of poly(methyl methacrylate) (PMMA) from a volatile solution of the polymer in toluene onto a solid substrate. We illustrate our experimental system in Figure 1, where in Figure 1a,b, we give a view from above and a side view of the microchamber and SAW device employed in the experiment. In Figure 1c, we demonstrate the integrated experimental system. The void in the microchamber is 7.5 mm wide, 4 mm long, and 75 μm thick. It consists of an underlying piezoelectric lithium niobate substrate and a glass cover above. The underlying substrate is a SAW device and may generate SAWs upon the application of power. The glass cover is endowed with high surface energy by employing hydrophobic coating. The resulting microchamber is open on one side. The opening is toward the source of the SAW, which is at the vicinity of electrodes fabricated on the lithium nioabate substrate—the SAW device.

**Figure 2:**
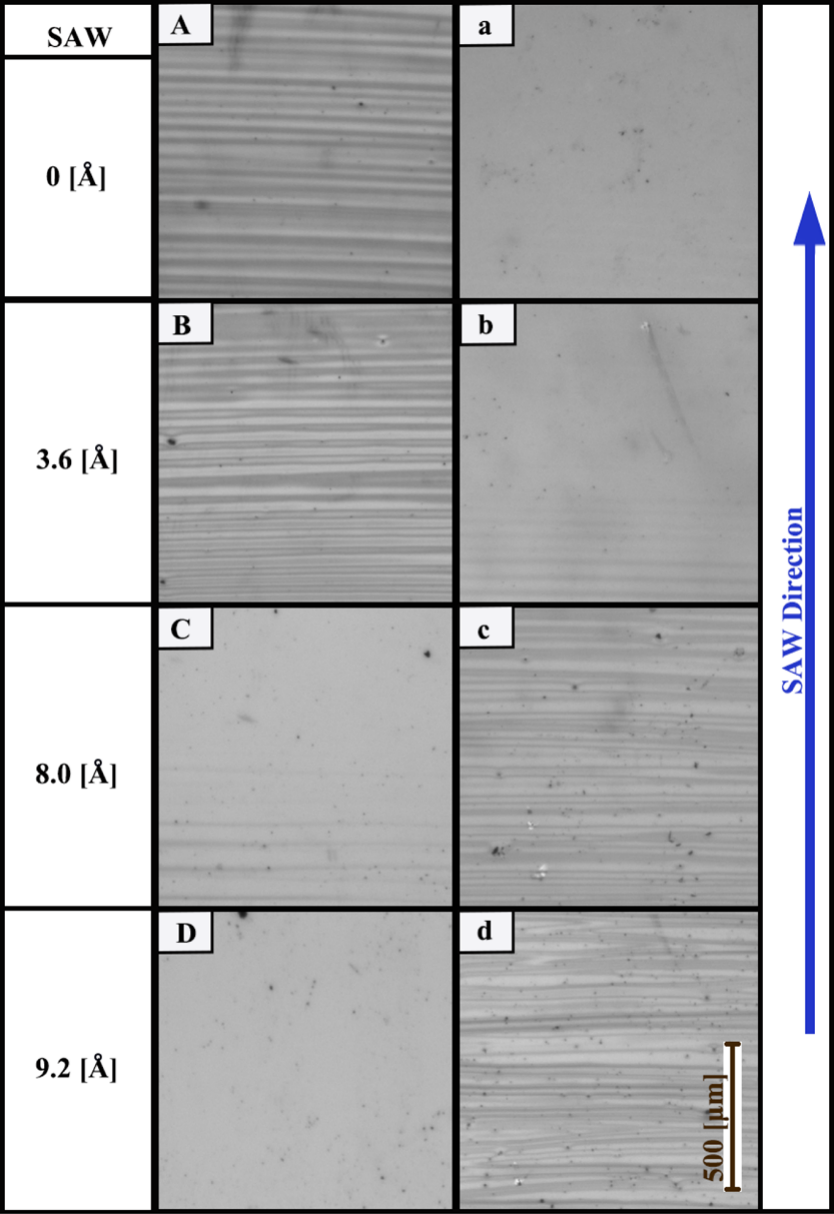
Sequences of top view images, which portray (A–D) surface of the underlying substrate, which may support a SAW, and (a–d) surface of an upper glass cover, following the full evaporation of a PMMA/toluene solution; the latter is initially confined between the two surfaces. The results are given for different levels of the SAW normal particle displacement amplitude (given in angstroms) at the surface of the underlying substrate, where the dark stripes are PMMA deposits and bright parts are devoid of deposits. The mass of polymer deposit on the underlying and upper substrates decreases and increases, respectively, with the increasing acoustic power (SAW particle displacement).

In Figure 2, we show the deposit of the PMMA polymer coating the underlying lithium niobate substrate and the glass cover of the microchamber following the full evaporation of the PMMA solution. The PMMA deposit appears as a stripe pattern; the stripes parallel the open side of the chamber. The formation of different types of deposit patterns is well known^22,23^ and is associated with the stick–slip motion of the three phase contact line, which is generated by an increase in viscosity of the polymer solution near the contact line during the evaporation of the solvent.^28,31,32^ Moreover, we observe that in the absence of SAWs, the PMMA deposit appears at the underlying lithium niobate substrate of the chamber and avoids the upper glass cover.

Previous studies^28,31^ indicated that polymer precipitates may favor coating some solid substrates over others when two different substrates are at proximity and in contact with a volatile polymer solution. In particular, they observed that polymer precipitates favor coating the substrate that supports the smaller three phase contact angle with the polymer solution. The connection between the magnitude of the three phase contact angle and enhanced or reduced coating of the substrate by polymer precipitates is not clear. However, the contact angle is a symptom of the free energy at the surface of a substrate. To identify the free surface energy of our substrates, we measured the corresponding advancing and receding contact angles at the three phase contact lines between the liquid, vapor, and solid substrates. On the hydrophobic [fluorine-doped tin oxide (FTO)-coated] glass, we measured advancing (θ_a_) and receding (θ_r_) contact angles of θ_a_ = 88 ± 2° and θ_r_ = 22 ± 2°, respectively, using DI water and θ_a_ = 13 ± 2° and vanishing contact angle values below the measurement capability of our goniometer (θ_r_ → 0), respectively, using toluene. On the lithium niobate surface, we measured advancing and receding contact angles of θ_a_ = 54 ± 2° and θ_r_ = 17 ± 2°, respectively, using DI water and θ_a_ = 12 ± 2° and vanishing contact angle values below the measurement capability of our goniometer (θ_r_ → 0), respectively, using toluene. The Young contact angle (θ_Y_) may be approximated to leading order using the rule^11,14^ cos θ_Y_ = (cos θ_a_ + cos θ_r_)/2. Hence, the Young contact angles for water and toluene on glass in our experiment are given by θ_Y_ = 61 ± 2° and θ_Y_ = 9 ± 2°, respectively. The Young contact angles for water and toluene on lithium niobate in our experiment are given by θ_Y_ = 39 ± 2° and θ_Y_ = 9 ± 2°, respectively. Using these measurements, we obtained^33,34^ that the free surface energies of glass and lithium niobate in our experiment are 0.041 ± 0.001 and 0.058 ± 0.001 N/m, respectively. The greater surface energy of lithium niobate—an oxide—gives a qualitative indication to the preference of the polymer precipitates to coat the lithium niobate substrate following the evaporation of the solution and in the absence of a SAW in our experiment.

The lithium niobate substrate in our experiment form the lower surface of our chamber. Hence, the preference of the polymer precipitates to coat the lithium niobate substrate in the absence of a SAW further raises a question about the contribution of gravity to the polymer deposition process. However, the contribution of gravity appears to be negligible when considering capillary-gravitational effects and polymer concentration distribution between the upper and lower parts of the chamber due to gravity. The former and latter gravitational contributions are associated with systems that exceed the capillary-gravitational length scale, given by , and the thermal gravitational length scale, given by *k*_B_*T*/*mg* ≈ 1 m, respectively, where γ = 28.5 mN/m, ρ = 0.87 g/mL, *g*, *k*_B_, *T* = 20 °C, and *m* = 350,000 Da are the surface tension and density of the polymer solution, gravitational acceleration, Boltzmann constant, temperature, and the mass of one polymer chain. Because the thickness of our chamber is 75 μm, it appears that the contribution of these two mechanisms to the polymer deposition process is small.

**Figure 3:**
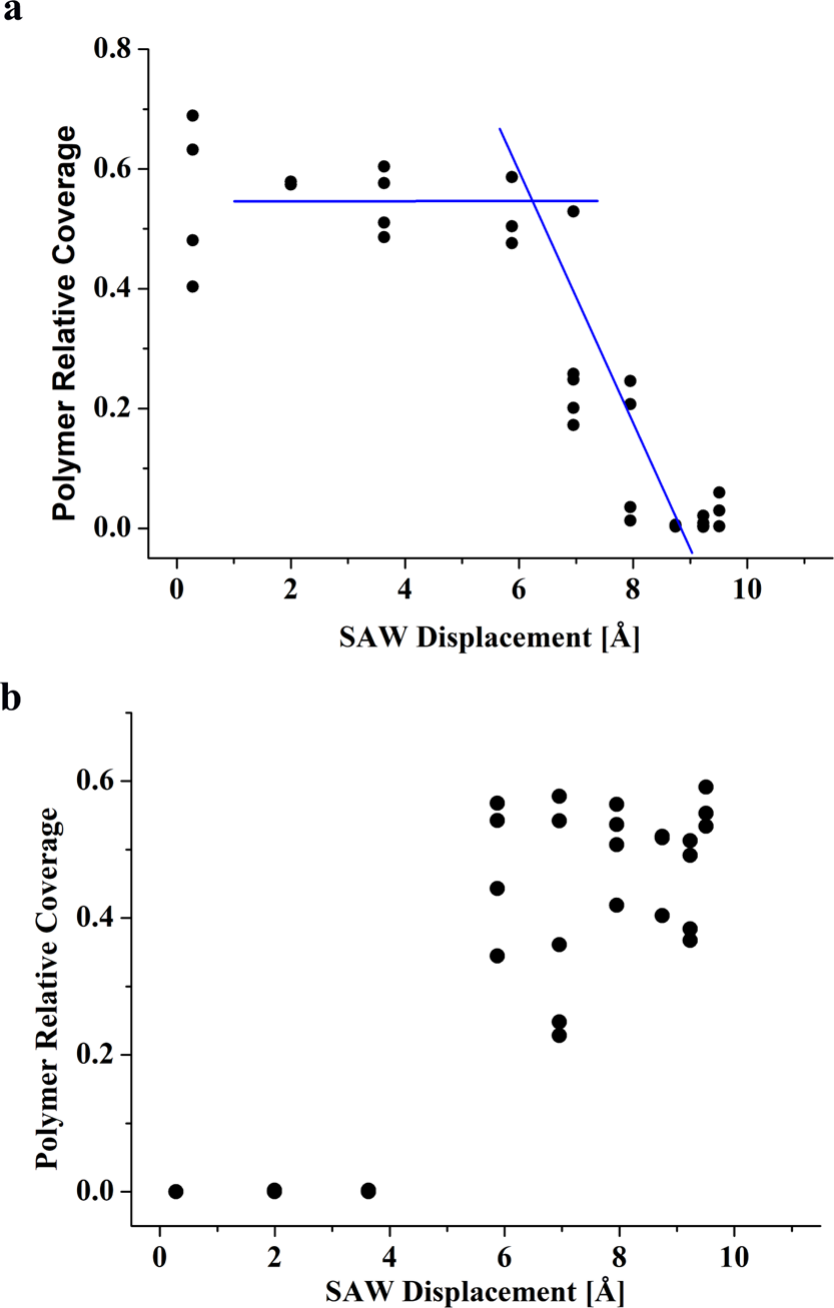
SAW particle velocity displacement variations of the relative coverage (dark parts divided by the full area of captured light microscopy images, as in Figure 2), atop (a) the underlying substrate of the chamber (which may support the SAW) and (b) the upper glass cover of the chamber. The solid blue lines are a guide to the eye. The characteristic error in the data obtained by image analysis is 10%.

When generating a propagating SAW in the underlying lithium niobate substrate, we observe that increasing the acoustic power inhibits the deposition of PMMA on its surface. Instead, PMMA precipitates appear to deposit on the hydrophobic cover glass. Above an acoustic power threshold, we do not observe PMMA deposits on the underlying lithium niobate substrate. In Figure 3, we give the ratio between the area covered by the polymer and the total area of the images obtained using light microscopy, quantifying the qualitative analysis shown in Figure 2. We employ an image analysis algorithm, which is given in detail in Supporting Information. The uncertainty of our results is approximately 10%, which mostly originates from the presence of impurities such as dust (black dots) in the microscopy images. A key observation shown in Figure 3 is an acoustic power threshold, which is manifested by a normal SAW particle displacement amplitude of approximately 0.6 nm (see the intersection between the two trend lines in the figure). Below the threshold, we observe that the patterned PMMA deposit covers approximately 40 to 70% of the underlying substrate. Above the power threshold (for values of particle displacement greater than 0.6 nm), we observe a sharp reduction in the area coverage of PMMA atop the substrate when increasing the acoustic power. When the SAW particle velocity is greater than 0.8 nm, we observe that the PMMA covers less than 10% of the substrate, which is within the margin of error of our image analysis. The PMMA cover ratio atop the hydrophobic glass gives an opposite trend to the one on the substrate of the SAW device. Below the power threshold, we do not find PMMA deposits atop the glass. Above the power threshold, we obtain a scatter of PMMA cover ratios, which indicate the presence of PMMA on glass.

**Figure 4:**
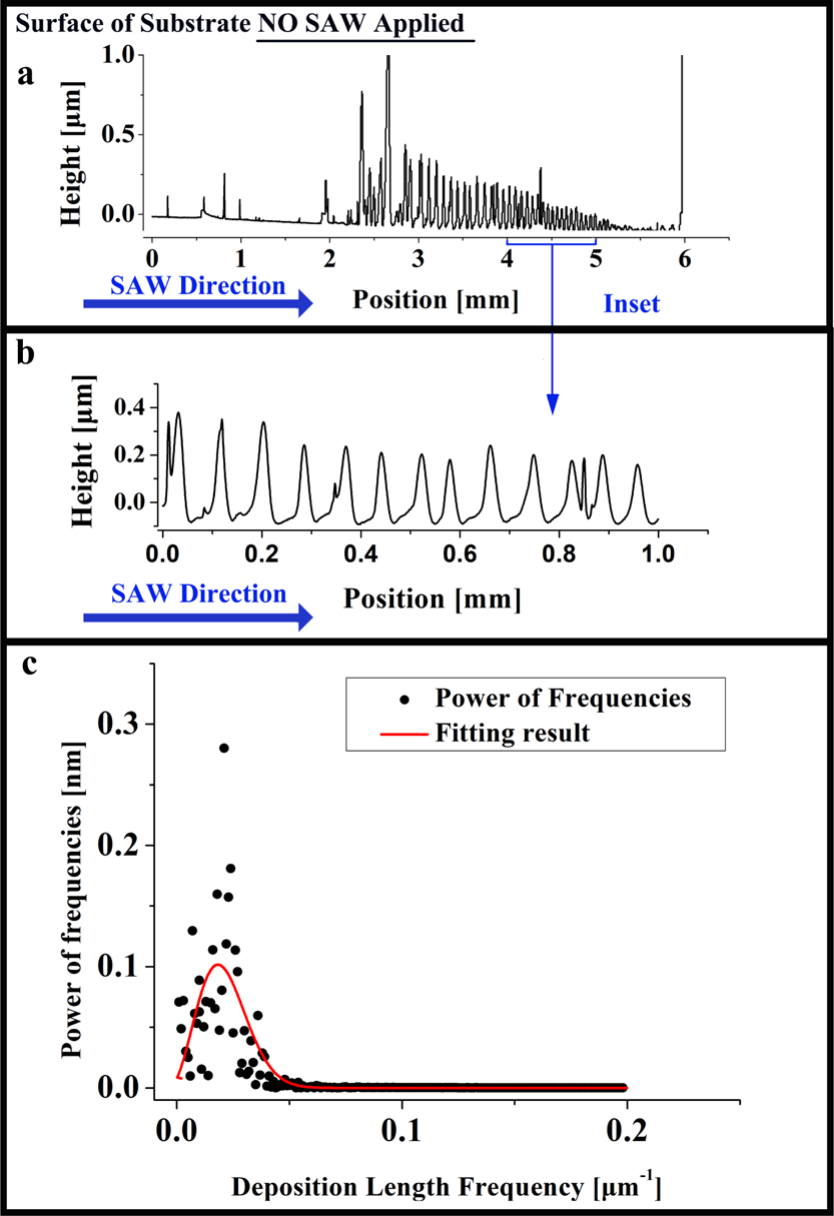
Illustration of capturing the characteristic length frequency of the stripe deposition of PMMA, where we show (a) the raw data of the stripe deposit (side view) obtained by profilometry, (b) part of the data analyzed, which is chosen far from the sides of the microchamber, and (c) a fast Fourier transform (FFT) of the data in image (b) (black dots) and a least square data fit to a corresponding distribution function (red solid line) whose maximum gives the characteristic frequency of the deposit (see Supporting Information).

**Figure 5:**
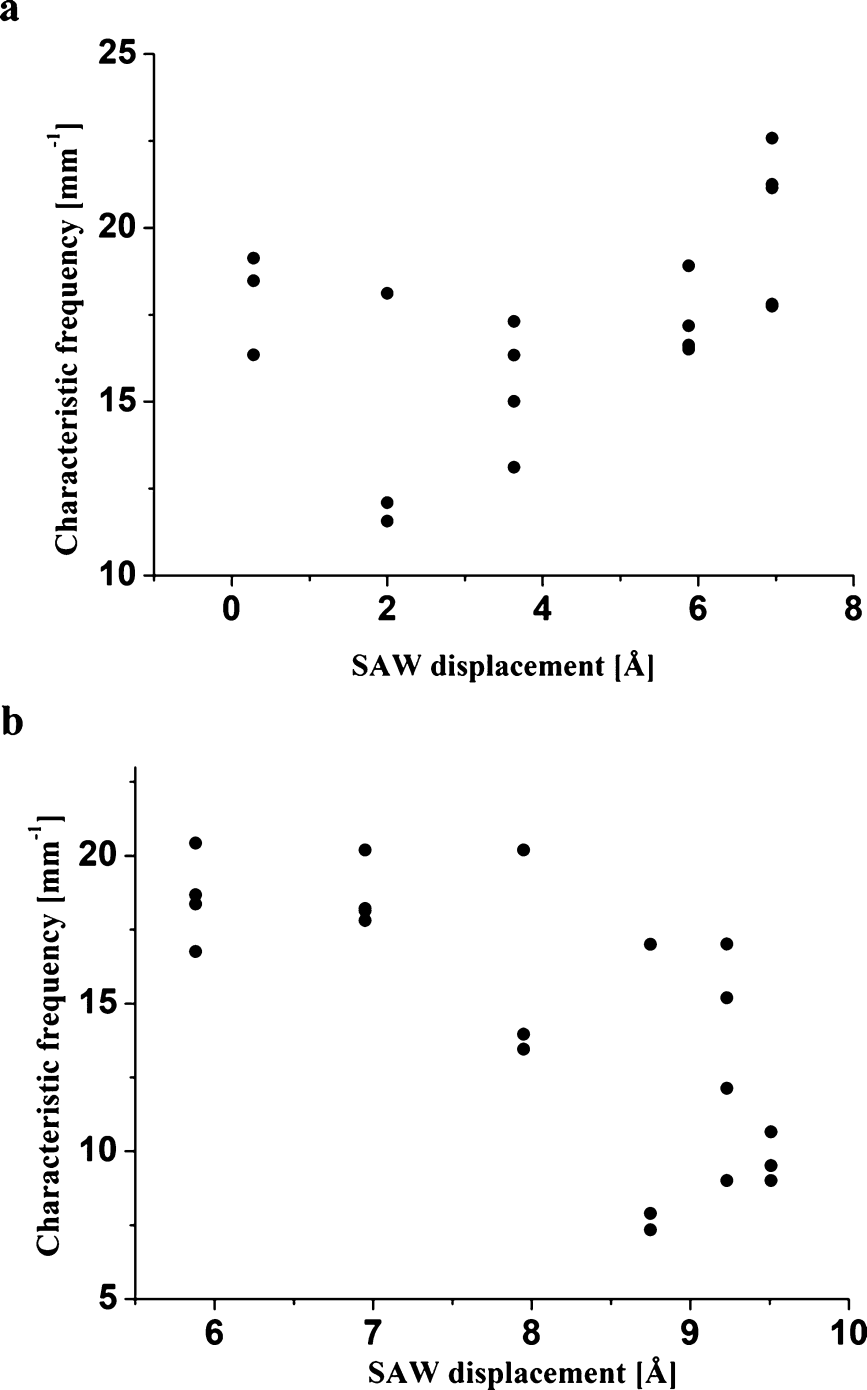
SAW particle velocity displacement variations of the characteristic spatial frequency of the stripes in the PMMA deposit, obtained according to the demonstration in Figure 4, atop (a) the underlying substrate of the chamber and (b) the upper glass cover of the chamber. The data for SAW particle displacement amplitude above and below 7 Å in (a) and (b), respectively, do not appear due to the luck of stripe-patterned PMMA deposits in these cases.

Figure 4a shows the profilometer measurement of the deposit morphologies. By employing FFT and a least square fitting of the Fourier transformed data to a skewed Gaussian function, we isolate the characteristic (or most pronounced) spatial frequency of the PMMA stripe deposits in each of our experiments. In Figure 5, we explore the sensitivity of the characteristic spatial frequency of the deposits to variations in the acoustic power (in terms of the particle velocity of the SAW). While the variance of our results increases with acoustic power, the measurements in the figures do not show any clear pattern. Hence, we observe that there is no clear indication that the spatial frequency of the stripe deposits changes with acoustic power.

## 3 Discussion and Conclusions

In this study, we demonstrate the application of a 20 MHz-frequency SAW in a lithium niobate substrate to mitigate the deposition of PMMA precipitates on its surface. In our experiment, we evaporate a polymer solution of PMMA in toluene within a 75 μm gap, which we fabricate between lithium niobate and hydrophobic glass substrates. In the absence of the SAW, we observe PMMA deposits atop the lithium niobate substrate, which supports greater free surface energy, following the full evaporation of the toluene solvent. The lower surface energy glass appears to remain devoid of PMMA deposits. The application of SAWs in the lithium niobate substrate inhibits the deposition of PMMA on the same substrate. Above an acoustic power threshold, we observe the absence of polymer deposits (within our error of measurement) on the lithium niobate substrate. Instead, we observe the deposition of PMMA on glass. The application of the SAW appears to actively prevent the deposition of PMMA on the substrate in which it propagates. Mass conservation requires the PMMA deposit to appear on glass. However, the SAW does not appear to have a clear contribution to the main spatial frequency of stripes in the PMMA deposits on lithium niobate and on glass, while the deposits are apparent on these substrates.

Our results are different to the ones obtained in previous studies on the application of SAWs in systems of convective self assembly. Mampallil et al.^29^ demonstrated that SAWs inhibit the pattern formation in the deposit (or the coffee ring effect) following the evaporation of a sessile drop of a volatile solution atop a piezoelectric lithium niobate substrate. Mhatre et al.^30^ demonstrated a similar observation by confining the liquid in a rectangular chamber, which was of similar geometry to the one employed here, albeit in their experiment, the internal thickness of the chamber was 1 mm (instead of 75 μm in the current study). It appears that in both the studies by Mampallil et al. and Mhatre et al., gravitational contributions to the deposition process were appreciable. The characteristic thickness of the drop in the former study was 1 mm, as was the thickness of the chamber in the latter study mentioned. Hence, the characteristic thickness of both systems was comparable in magnitude to the gravitational-capillary length scale of 2 mm. While in the work by Mampallil et al., there was only one underlying lithium niobate substrate that supported a SAW, and in the work of Mhatre et al., there were two substrates, one of which was lithium niobate that supported a SAW and the other was inert glass; it appears that in both experiments, the deposition of precipitating solute mass continued to take place on the underlying lithium niobate surfaces that supported the SAW. Hence, the application of SAWs altered the morphology of the deposit on the lithium niobate substrates, but did not eliminate the deposits. In particular, both the studies by Mampallil et al. and Mhatre et al. reported that the originally patterned deposits (in the absence of SAWs) were altered to homogeneous coatings in the presence of SAWs. Here, we show that given a small enough separation between two solid substrates that confine a volatile solution, it appears that the presence of a propagating SAW in one of the substrates precludes the coating of the latter by polymer precipitates. The polymer will coat the opposing inert substrate.

The presence of the SAW gives an active mean to avoid mass deposition on sensitive substrates by employing nearby sacrificial surfaces, which serve as sinks to the deposit. We believe that our insight about the mitigation of precipitate deposition on a substrate that supports a propagating SAW may appear useful to replace the necessity for cleaning unwanted deposits and corresponding maintenance in systems whose parts come in contact with volatile solvents or solvents that undergo temperature change, which alter the content of the dissolved solute. Moreover, employing the SAW for active self-cleaning surfaces may be preferred over passive self-cleaning surfaces in many application due to the absence of the requirement for a rough surface in the former. Examples are domestic utilities and heat exchangers, especially in delicate systems such as in the case of liquid cooling for high power electronics with or without phase change and in the case of microfluidic platforms that process volatile liquids. Generally, the SAW will propagate also in solids that support rough surfaces, albeit the roughness should be much smaller than the wavelength of the SAW to minimize the interference between the roughness and the SAW. Moreover, the SAW will propagate through smooth curvatures at the substrate and will be able to transport through sharp (90°) corners subject to a loss of approximately 50% of the local acoustic power following each corner. However, in the latter case, one must be cautious due to the reflection of part of the SAW from the vicinity of the corner, which may result in a partially standing SAWs with unclear implications to the precipitate deposition process.

## 4 Experimental Methods

Our microchamber shown in Figure 1 is composed of a glass cover, an underlying substrate that may support SAWs, and a spacer. The glass cover is rendered hydrophobic by a coating of FTO (TISX 001, Techinstro). The manufacturer specification for the surface roughness of the hydrophobic glass is approximately 1–10 nm. The underlying substrate is a SAW device, which is composed of 5 nm titanium/1 μm aluminum interdigitated electrodes, patterned using standard lift-off photolithography atop a 0.5 mm thick, 128° *Y*-cut, *X*-propagating, single crystal lithium niobate (LiNbO_3_) piezoelectric substrate. The manufacturer specification for the surface roughness of the lithium niobate substrate is approximately 1 nm.^35^ The two substrates are separated by a 75 μm thick gap using a polyimide (Kapton, DuPont) “U”-shaped spacer. The resulting microchamber is open on one side. The opening is toward the source of the propagating SAW on the SAW device (the electrodes). To confine the SAW device and connect the latter to power, we 3D-printed an elastomeric stage (shown in Figure 1c). Using pogo pins (BC201403AD, Interconnect Devices, INC.), we connected the electrodes on the actuator to a signal generator (R&S SMB100A microwave signal generator) and an amplifier (model A10150, Tabor Electronics Ltd.). The SAW is generated by imposing different levels of the continuous sinusoidal voltage input with a fixed frequency of 20 MHz. Using a scanning laser Doppler vibrometer (MSA-500, Polytech), we measured the corresponding normal displacement amplitude (particle displacement of the SAW) over a surface area of approximately 1 × 1 mm^2^ near the electrodes and verified that the SAW is a propagating wave (see Supporting Information). The displacement may be translated to a corresponding normal particle velocity of the surface by multiplying the given displacement amplitudes by 2π × 20 MHz. We further used a goniometer (Data Physics; OCA 15Pro) to measure the three phase contact angles of the liquids with the substrates employed.

At the beginning of our experiments, we injected a solution of PMMA (average *M*_w_ 350,000 by GPC, 9011-14-7, Sigma-Aldrich) and toluene (AR-b, 99.7%, 108-88-3, Bio-Lab Ltd.) into the chamber through its open side. We employed a solution concentration of 10 mg/mL, which gives consistent patterns for same physical parameters.^28^ The ambient temperature and relative humidity in the laboratory were set at approximately 20 °C and 50% using a dedicated air conditioning system. We further employed a hot plate (DBD-001, MRC Ltd-Laboratory Equipment) to maintain our experimental system at a temperature of 35 ± 1 °C, which we measured using an infrared thermometer (RAYMT4U, Raytek) near the opening of the microchamber. Prior to every single experiment, we first employed toluene to rinse the different parts of the disassembled system. Then, the parts were fully submerged in toluene in an ultrasonic cleaner (AC-200H, MRC Ltd-Laboratory Equipment). Next, we rinsed every part for 30 s by 5 different solvents in the following order: toluene (AR-b, 99.7%, 108-88-3, Bio-Lab Ltd.), acetone (AR-b, 99.8%, 67-64-1, Bio-Lab Ltd.), 2-propanol (AR-b, 99.8%, 67-63-0, Bio-Lab Ltd.), ethanol (CP-p, 96%, 64-17-5, Bio-Lab Ltd.), and water (HPLC plus, 7732-18-5, Sigma-Aldrich). Finally, we dried the different parts using air flow.

Prior to measurement, we assembled the system in the manner given in Figure 1. Every test started by measuring the rate of solvent evaporation to assess the integrity of the microchamber. We injected 10 μL of pure toluene into the microchamber and observed the motion of the meniscus of the volatile liquid. We further verified the presence of SAWs prior to each experiment by positing a single drop of toluene atop the SAW device and monitored the displacement of the drop under SAW excitation. Following these assessments, we let the SAW microfluidic platform rest for 20 min prior to the actual experiments to facilitate the full evaporation of the pure toluene.

During each measurement, we kept the different conditions constant, except for the voltage applied from the signal generator. In our measurements, we first generated the SAW, and then, we injected the microchamber with 10 μL PMMA/toluene solution, rendering the microchamber devoid of bubbles. The evaporation of toluene lasted for several minutes during the measurement. At the end of the experiment, we arrested the generation of the SAW and disassembled the platform 25 min following the observation that the meniscus of the solution has reached the end of the chamber. We employed this precaution because residual solution, if remained near the sides/corners of the chamber, was found to destroy the deposit upon disassembling the chamber components.

After disassembling the platforms, we measured the two surfaces in question (upper cover and underlying substrate) using light microscopy (Eclipse Ni-E, Nikon) and a profilometer (Dektak, Bruker). Overall, we employed nine different unique power levels of the SAW in our experiments. For each unique level, we performed at least three repeated experiments for assessing the consistency of results. The analysis of the experimental data was conducted by using an “observation window” at an area of 1 mm × 1 mm in the middle of microchamber, 1 and 2 mm away from the rear and front (open) sides of the chamber, respectively, to avoid possible side effects.

## Supporting Information Available

Calibration of applied voltage versus SAW displacement, image analysis, and frequency analysis of microtopographic data (PDF)
