# Supplementary material for: Surface Acoustic Wave Mitigation of Precipitate Deposition on a Solid Surface—An Active Self-Cleaning Strategy
Source: ACS Appl Mater Interfaces. 2021 Dec 1;13(49):59471–7. doi: 10.1021/acsami.1c17778 (PMC8678987; doi:10.1021/acsami.1c17778)
Supplement: Supplementary file 1 — am1c17778_si_001.pdf [file am1c17778_si_001.pdf]

Surface Acoustic Wave Mitigation of Precipitate Deposition on a Solid Surface – An Active Self-Cleaning Strategy

Yifan Li,[†] Dario R. Dekel,[*,†,‡] and Ofer Manor[*,†]

The Wolfson Faculty Department of Chemical Engineering, Technion - Israel Institute of Technology, 3200003 Haifa, Israel, and The Nancy & Stephen Grand Technion Energy Program(GTEP), Technion Israel Institute of Technology, Haifa 3200003, Israel

E-mail: dario@technion.ac.il; manoro@technion.ac.il

Supporting Information

[*]To whom correspondence should be addressed
[†]Technion - Israel Institute of Technology
[‡]The Nancy & Stephen Grand Technion Energy Program

Calibration of applied voltage versus SAW displacement

Below we give a calibration plot of the applied voltage versus the average RMS SAW displacement measured on the SAW device over a surface area of approximately 1×1 mm^2 near the electrodes on the SAW device.

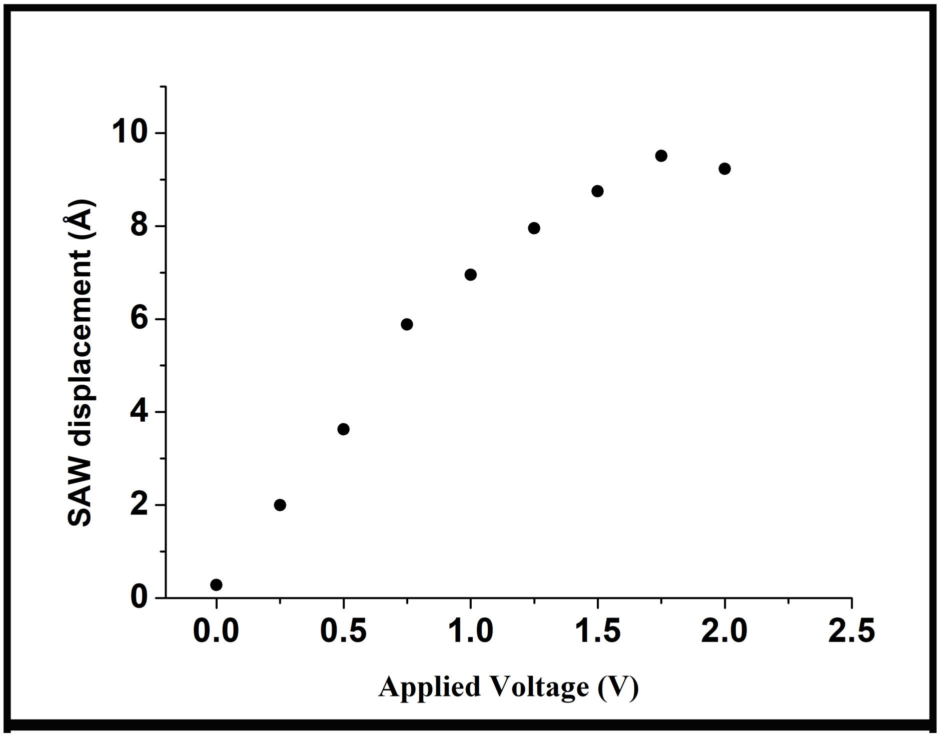

Figure 1: Applied voltage variations of the average normal SAW displacement at the surface of our SAW device

Image analysis

For every single experiment, two images from the microscope were used for analysis, focusing on the center of the two surfaces in question. (cover and substrate) The ratio between pixels and length was calculated by ImageJ. The images were used for calculating the cover ratio in figure 3 by a self-written code in MATLAB. The algorithm firstly rotated the image at an angle range of $-10°$ to $10°$. For each rotated image, the average value of the gray value (Gray value represents the brightness of the image) of each row was collected as a new array. Notably, if the image is not rotated, we recieved an array with the size of 310. And if the

image is rotated, the size decreases because of the shrinkage of rotation. Then the standard deviation of the elements in the array was calculated. For the picture with stripes that parallel to the top and bottom edge of the image, a greater deviation is expected. We call this deviation value parallel factor. The rotated image with the largest parallel factor was chosen because they possess the best performance of showing stripes in parallel.

The next parameters we calculated for the processed images are Otsus threshold.[1] The threshold is a number in the range of gray values of the image and it is used for filtering the original images into a binary color scheme. Otsus method separates the range of gray value histogram into two sections and minimizes the intra-class variance of those sections. The intra-class variance $\sigma_w(t)$, indicating the performance of separation, is defined as $\sigma_w^2(t) = \omega_0^2(t)\sigma_0^2(t)+\omega_1^2(t)\sigma_1^2(t)$, where $\sigma_1(t)$ and $\sigma_0(t)$ are the sum of probabilities (integral of probabilities) of two sections and $\omega_1^2(t)$ and $\omega_0^2(t)$ are the variances of these sections. This algorithm exhaustively goes over all the possible t (the value of threshold) and finds the minimal $\sigma_w(t)$ and corresponding t_f as the final threshold. By filtering the images with their corresponding t_f, which means changing the pixels value above t_f as 1 (white) and below as 0 (dark), we transfer the images into binary images, where one color gives polymer coverage and the other color gives the bare surface of substrate.

Before we calculated the cover ratio from the binary images, one more check was applied. We divided the images into four separated and overlapped parts and calculated the average gray value of the different parts. We compared the ratio between the sum of the average gray values between the different parts of each figure. If the ratio value was found greater than 0.5 (based on comparison between surfaces with polymer deposits and surfaces without polymer deposits) we avoid the quantitative analysis of the corresponding image. Where our images passed this text, t we calculated the polymer cover ratio $Ratio = S_0/S_{total}$, where S_0 is the total number of pixels with a value of 0 (dark, representing polymer) and S_{total} is the total number of pixels in the image.

Frequency Analysis of Microtopographic Data

We utilized microtopographic data by profilometer in the observation window (Line of interest) and summarized the characteristic properties out of data by MATLAB code written by us.

Following the transformation of the profilometer data to the frequency domain using FFT, we obtained a discrete power spectrum of the different frequency in the partnered stripe deposits. The maximum power in the FFT spectrum represent the most common repeating wavelength in the deposit.

To assess the most common frequnecy in the FFT power spectrum, we employed least square fit of the latter to the distribution function

$$y = p_1(x - p_3)^2 \exp\left(\frac{-(x - p_3)^2}{(2p_2^2)}\right) \qquad (x > p_3)$$

$$y = 0 \qquad (x < p_3)$$

where p_1, p_2, p_3 are three fitting parameters. The spatial frequency that gives the maximum power value in the distribution function is taken to be the main repeating spatial frequency in the analyzed striped deposit.

References

(1) Otsu, N.; Smith, P. L.; Reid, D. B.; Environment, C.; Palo, L.; Alto, P.; Smith, P. L. Otsu_1979_otsu_method. *IEEE Trans. Syst. Man Cybern.* **1979**, *C*, 62–66.
